# Integrating multimodal features to predict the malignancy of pulmonary ground−glass nodules: a multicenter prospective model development and validation study

**DOI:** 10.3389/fonc.2025.1547816

**Published:** 2025-03-21

**Authors:** Yuanhui Wei, Wei Zhao, Zhen Wu, Nannan Guo, Miaoyu Wang, Hang Yu, Zirui Wang, Wenjia Shi, Xiuqing Ma, Chunsun Li, Jiabo Ren, Yue Yin, Shangshu Liu, Zhen Yang, Liang-an Chen

**Affiliations:** ^1^ School of Medicine, Nankai University, Tianjin, China; ^2^ Department of Respiratory and Critical Care Medicine, Eighth Medical Center, Chinese PLA General Hospital, Beijing, China; ^3^ Department of Thoracic Surgery, Fourth Medical Center, Chinese PLA General Hospital, Beijing, China; ^4^ Medical School of Chinese People’s Liberation Army, Beijing, China; ^5^ Department of Respiratory and Critical Care Medicine, Beijing Northern Medical District, Chinese PLA General Hospital, Beijing, China

**Keywords:** lung cancer, ground-glass nodule (GGN), predictive model, multimodality, radiomics, triosephosphate isomerase-1, MicroRNA-206

## Abstract

**Background:**

There is a clinical need for accurate noninvasive evaluation of the malignancy of pulmonary ground−glass nodules (GGNs) to reduce risks of overdiagnosis and overtreatment. This study aimed to develop and validate a clinic-biomarker-combined deep radiomic model for the prediction of GGN malignancy.

**Materials and methods:**

This study recruited patients with GGNs from seven medical centers across five cities in China. The participants included in this study were divided into the training-validation and the test groups on the basis of the centers from which they were recruited. The malignancy of GGNs was determined based on pathological results. Clinical, radiological, and biomarker features with significant differences were used to establish predictive models. Six types of models based on different features were developed on the training-validation group: clinical-radiological (CR), biomarker-combined CR (B-CR), deep radiomic (DR), clinic-combined DR (C-DR), biomarker-combined DR (B-DR), and clinic-biomarker-combined DR (CB-DR) models. The models were then evaluated on the test group for discrimination, calibration, and clinical utility.

**Results:**

A total of 501 participants with 571 GGNs were included in the study. Four hundred and seven participants with 454 GGNs were assigned to the training-validation group, whereas 94 participants with 117 GGNs were assigned to the test group. Significant differences were observed in sex, smoking history, triosephosphate isomerase-1 and microRNA-206 between patients with and without malignant GGNs. And size, location, and lobulation were significantly different between benign and malignant GGNs. Among all the models, the CB-DR model achieved the highest performance in classifying GGNs, with an AUC of 0.90 (95% CI: 0.81-0.97). At the optimal cutoff, the corresponding accuracy, sensitivity, and specificity were 0.89 (95% CI: 0.83–0.94), 0.90 (95% CI: 0.84–0.96), and 0.82 (95% CI: 0.62–1.00), respectively. Furthermore, malignancy evaluation based on the CB-DR model would have reduced overtreatment for 82.4% (14/17) of benign GGNs and enabled timely interventions for 90.0% (90/100) of malignant GGNs.

**Conclusion:**

The CB-DR model developed in this study exhibited satisfactory performance in predicting the malignancy of GGNs and holds potential as a valuable tool for aiding clinical decision-making in GGN management.

## Introduction

Lung cancer remains the leading cause of cancer-related death worldwide (1). Early detection through CT screening has significantly improved patient prognosis and reduced mortality rates (2). In screening and incidental detection, an ever-growing number of pulmonary ground−glass (GGNs) are identified (3, 4). Consequently, the management of GGNs has become a critical concern in clinical practice. GGNs present as hazy opacities on CT scans that do not obscure underlying structures, such as blood vessels and bronchi (5). GGNs can be further classified into pure GGNs (pGGNs) and mixed GGNs (mGGNs) based on the absence or presence of solid components. In comparison to solid nodules, GGNs are associated with an increased risk of malignancy (4, 6, 7). However, most lung cancers manifesting as GGNs stay in the early stage of the disease, exhibit relatively favorable prognoses, and can be treated with minimally invasive interventions (8–10). The natural progression of malignant GGNs involves a transition from pGGNs to mGGNs, and finally to solid nodules (11). As such, the GGN stage represents the optimal window for lung cancer intervention. Nonetheless, GGNs are not exclusive to lung cancer. Many benign lesions, such as infections and inflammation, also appear as GGNs (12). Benign GGNs typically do not necessitate invasive interventions. Therefore, accurate classification of benign and malignant GGNs is a critical issue in the management of GGNs.

Currently, evaluating GGNs relies primarily on radiological assessments and follow-up observations (5). However, owing to the overlapping radiological characteristics of benign and malignant GGNs, distinguishing them is challenging, which leads significant risks of overdiagnosis and overtreatment. Moreover, long-term CT follow-ups contribute to increased radiation exposure, heightened patient anxiety, and elevated healthcare costs (13, 14).

Radiomics, a methodology that extracts extensive image features through high-throughput computational analysis of voxel distribution patterns, has demonstrated great potential in addressing these challenges (15). Recently, deep radiomics, which is based on deep learning techniques such as convolutional neural networks (CNNs), has yielded promising results in medical imaging analysis. Compared with conventional handcrafted radiomics, which relies on predefined image analysis theories, deep radiomics adaptively extracts image features and offers substantial potential for accurately identifying malignant pulmonary nodules (16). For example, Liu et al. developed an attention-gated CNN model to classify pulmonary nodules (17). The model achieved a high AUC of 0.89.

To further improve accuracy, biomarkers have emerged as important tools (18). Liang et al. developed a predictive model based on DNA methylation for malignant lung nodules, the PulmoSeek (19). Building on this model, He et al. developed a combined model of clinic, radiology, and DNA methylation biomarkers, the PulmoSeek Plus, which showed improved performance and greater potential for aiding in the early diagnosis of lung cancer (20).

Despite considerable advances in deep radiomics and biomarkers, satisfactory results have yet to be achieved in the prediction of the malignancy of GGNs. We believe that a multimodal analysis of clinical features, biomarkers, and radiomics could improve the evaluation of GGNs. This study aimed to develop and validate a clinic-biomarker-combined deep radiomic model to predict the malignancy of GGNs.

## Materials and methods

### Participant recruitment and grouping

This was a multicenter prospective model development and validation study, which was approved by the ethics committee of Chinese PLA General Hospital (S2020-173-01) and then registered at ChiCTR.org.cn (ChiCTR2100044576).

**Figure 1:**
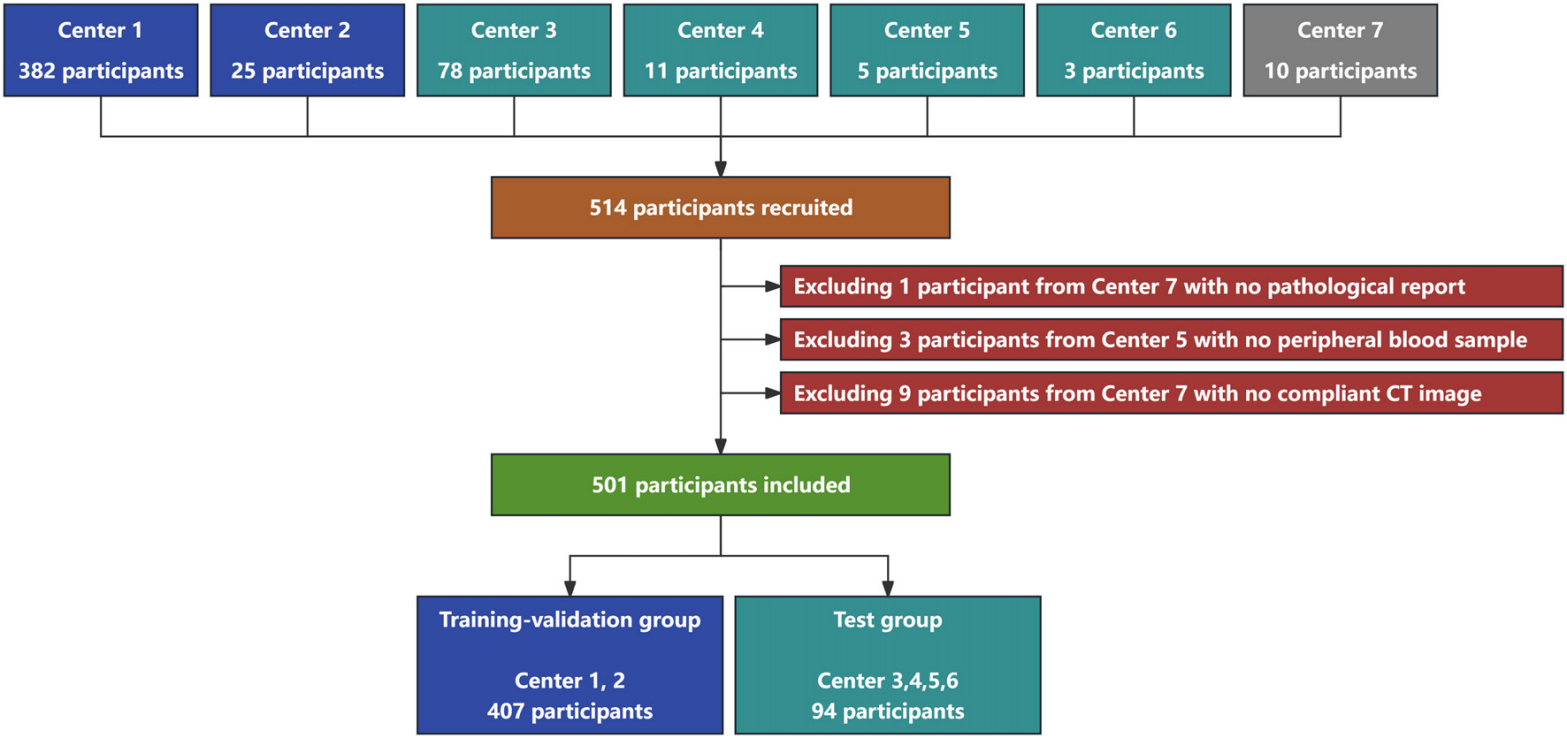
Flowchart of participant recruitment and grouping in this study. A total of 514 participants were recruited from seven medical centers. Thirteen participants were excluded, and 501 participants with 571 GGNs were finally included in this study. Four hundred and seven participants with 454 GGNs were assigned to the training-validation group, whereas 94 participants with 117 GGNs were assigned to the test group.

The participants were recruited between June 2021 and May 2023 from seven medical centers across five cities in China (**Figure 1**). The involved centers included: the First Medical Center of the PLA General Hospital (Center 1), the Qinhuangdao People’s Hospital (Center 2), the Fourth Medical Center of the PLA General Hospital (Center 3), the Second Affiliated Hospital of Dalian Medical University (Center 4), the Second Affiliated Hospital of the Army Medical University (Center 5), the Sixth Medical Center of the PLA General Hospital (Center 6), and the Qingdao Municipal Hospital (Center 7). The participant inclusion criteria were as follows (1): patients or their legal representatives consented to participate and signed informed consent; (2) patients were aged between 18 and 80 years; (3) patients with clinically detected GGNs; and (4) patients were scheduled for minimally invasive biopsy or surgical intervention. The participant exclusion criteria were as follows: (1) participants with no pathological report; (2) participants with no peripheral blood sample or lung CT image with a slice thickness ≤1.5 mm; (3) participants with a history of lung cancer; (4) participants who withdrew from the study; and (5) participants with poor compliance.

The participants included in this study were divided into the training-validation and the test groups on the basis of the center from which they were recruited. The participants from centers 1 and 2 were assigned to the training-validation group, whereas those from centers 3, 4, 5, and 6 were assigned to the test group.

Participant information was entered and uploaded through a secure verification-required website. And the malignancy of GGNs was determined based on pathological results.

### CT image acquisition and blood sample collection

Prior to initial treatment, CT imaging and blood sample collection were conducted.

CT scans were performed using SOMATOM Definition Edge (Siemens AG, Germany), LightSpeed Volume CT (General Electric Company, USA), and Brilliance iCT (Royal Philips, Netherlands) scanners. The tube voltage was generally set at either 100 or 120 kVp, with the tube current adjusted automatically or manually based on patient-specific factors. The reconstruction matrix was set at 512 × 512, with a slice thickness of 1.0–1.5 mm and a pixel interval of 0.5–0.8 mm. CT images from other centers were shipped to the Big Data Research Laboratory of the Department of Pulmonary and Critical Care Medicine at Chinese PLA General Hospital using encrypted USB drives. All the CT images acquired for the study were unenhanced.

Blood samples were drawn in the morning after an overnight fast to minimize variations in blood metabolites. Venipuncture was performed by trained nursing staff at each center using sterile techniques. Whole blood samples were collected in EDTA tubes and gently inverted to mix with the anticoagulant. For the serum samples, blood was collected in plain tubes, allowed to clot at room temperature for 30 minutes, and then centrifuged at 3000 × g for 10 minutes at 4°C. Following preprocessing, samples from other centers were shipped to the Laboratory of the Department of Pulmonary and Critical Care Medicine at Chinese PLA General Hospital and immediately stored at -80°C until analysis.

### GGN segmentation and image preprocessing

The GGNs were manually segmented by a senior pulmonologist via 3D Slicer (version 5.6.1; slicer.org). The pulmonologist was blinded to the pathological results of GGNs.

The voxel spacing of the CT images was reconstructed into 1.0 × 1.0 × 1.0 mm. The image patch with 64 × 64 × 64 voxels was cropped from the reconstructed CT image. CT values were converted to density values using Equation 1. Density values less than 0 were clipped to a minimum of 0.


(1) $$\text{Density values }=\;\frac{\text{CT values }+1000}{1000}$$

### Biomarker measurement

In our previous unpublished study, we conducted multi-omics analysis to compare the molecular expression profiles of participants with malignant GGNs before and after surgery. Triosephosphate isomerase-1 (TPI-1) and microRNA-206 (miR-206) were identified as promising biomarkers for predicting the malignancy GGNs. The current study assessed the value of combining these biomarkers for classifying GGNs.

The procedure for measuring the serum TPI-1 concentration was conducted briefly as follows: prewashed magnetic beads were added to the serum to enrich low-abundance proteins. After incubation, the supernatant was discarded, and the magnetic beads were washed. The magnetic beads were placed in urea lysis buffer to lyse and release the bound proteins. Dithiothreitol (DTT) was added to reduce disulfide bonds, and iodoacetamide (IAA) was added to alkylate cysteine residues. Following centrifugation at 10,000 × g for 5 minutes, the supernatant was collected, and the proteins were digested with trypsin. The resulting peptides were desalted via a C18 solid-phase extraction column (Sigma, USA). The peptide concentrations were quantified by measuring the ultraviolet absorbance at 280 nm using a NanoDrop 2000 spectrophotometer (Thermo Fisher Scientific, USA). Indexed retention time (iRT) calibration peptides were spiked into each sample. Peptides from each sample were analyzed using a timsTOF mass spectrometer (Bruker, USA) coupled to an Evosep One liquid chromatography platform (Evosep, Denmark) in data-independent acquisition (DIA) mode. The DIA data were analyzed using Spectronaut 16 (Biognosys, Switzerland).

The relative expression of miR-206 in the blood was quantified according to the manufacturer’s instructions. Briefly, total RNA was extracted from blood using TRIzol LS reagent (Thermo Fisher Scientific, USA). The extracted miRNA was reverse transcribed into cDNA using the miRcute Plus miRNA First-Strand cDNA Kit (TIANGEN BIOTECH, China). qRT−PCR was performed using the KAPA SYBR FAST qPCR Kit (Roche, Switzerland). The relative expression of miR-206 was calculated by the ΔCT method with U6 as the endogenous control (Ct-RNA minus Ct-U6). The primers for miR-206 were CAACACAATGGAATGTAAGGAAGT and TATGGTTGTTCTGCTCTCTGTCTC. The primers for U6 were GCTTCGGCAGCACATATACTAAAT and CGCTTCACGAATTTGCGTCTCAT.

### Model development

PyCaret (version 3.3.2; pycaret.org) was used to automate the development of clinical-radiological models. Six machine learning algorithms were used to construct models: logistic regression (LR), support vector machine (SVM), decision tree (DT), random forest (RF), extreme gradient boosting (XGBoost), and light gradient boosting machine (LightGBM).

Deep radiomic models were built based on RegNet, which is a CNN optimized by neural architecture search (NAS) and has better performance than ResNet (21). To accommodate CT images, a three-dimensional version of RegNet was used in this study. Three RegNet models with different complexity levels of 4, 16, and 64 were selected as candidates, and the corresponding model structure parameters were derived from the original Y-400MF, Y-1.6GF, and Y-6.4GF RegNet models, respectively. In addition to the CT image, the distance map was innovatively used as additional input to guide the models to learn the concepts of locations and regions. The distance map is a map that marks the signed distance from each voxel point to the nodule surface (negative inside and positive outside), which is computed by SimpleITK (version 2.3.1; simpleitk.org). For the combined models, the additional clinical features and/or biomarkers were concatenated with deep radiomic features for prediction (**Supplementary Figure 2**).

Conventional methods, such as Gaussian noise, random cropping, minority oversampling, and class weighting, were used to compensate for the limited amount of data and the imbalanced distribution of classes. Additionally, the manifold mixup technique was employed to enhance the abilities of the deep radiomic models to learn more effective representations. This technique generates new samples by linearly interpolating both the extracted features and the corresponding labels of raw samples, which may help models learn smoother and more robust representations (22).

To enable the deep radiomic models to acquire foundational radiological knowledge, the models were pretrained on the Lung Nodule Analysis 16 (LUNA16) dataset, a subset of the Lung Image Database Consortium and Image Database Resource Initiative (LIDC-IDRI) dataset (23). The dataset includes 1,186 nodules and the corresponding annotations provided by four radiologists. The pretraining task involved predicting nodule attributes such as margin, lobulation, spiculation, and malignancy scores (see **Supplementary Table 1** for the results).

Besides, the class activation mapping (CAM) was used to visualize the decision-making process of the deep radiomic models (24). And the deep radiomic models used in this study were implemented with the PyTorch framework (version 2.3.1; pytorch.org).

**Figure 2:**
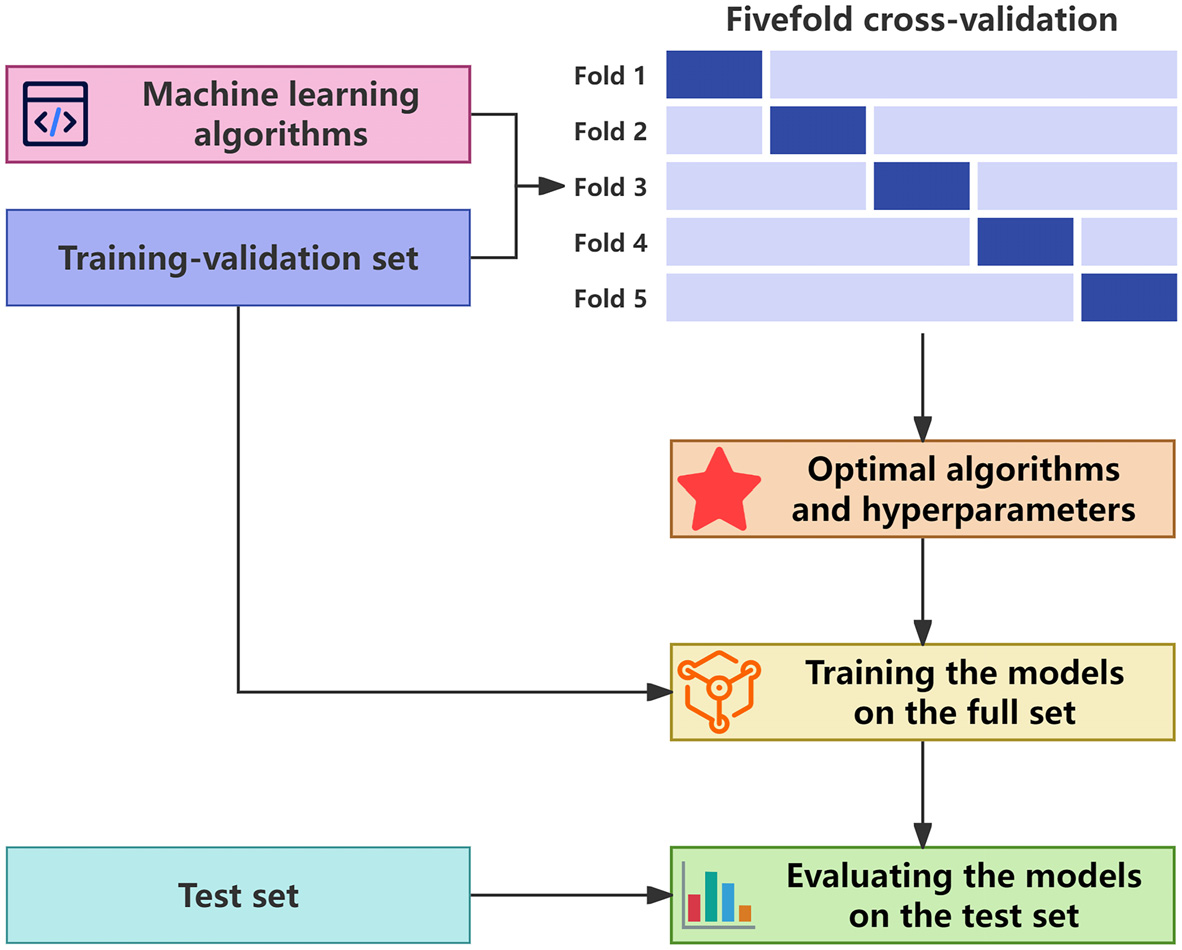
Workflow of model development and evaluation. The optimal algorithms and hyperparameters for each model type were determined in fivefold cross-validation on the training-validation set. The optimal models were then trained on the complete training-validation set and evaluated on the test set.

Fivefold cross-validation was performed on the training-validation set to determine the optimal algorithms and hyperparameters for each model type. The optimal models were then trained on the full training-validation set and evaluated on the test set (**Figure 2**).

### Model calibration

The models were calibrated via isotonic regression with fivefold cross-validation on the training-validation set. The calibrated result was computed by averaging the outputs from all five folds. The final prediction was adjusted by Equation 2, where ω represents the proportion of benign GGNs in the training-validation set. This adjustment was applied to mitigate the impact of class imbalance (25).


(2) $$p_{adjusted}=\frac{p_{raw}}{p_{raw}+\frac{1-p_{raw}}{\omega }}$$

### Statistical analysis

Statistical analyses were conducted using R (version 4.4.1; r-project.org) and Python (version 3.11.5; python.org). Categorical variables were compared between groups using the chi-square test. For continuous variables, the Student’s t-test was applied when the assumption of normality was met; otherwise, the Kruskal-Wallis test was used. Differences between model predictions and observed outcomes were assessed with the Spiegelhalter’s z-test. The incremental predictive values of the models were assessed using the net reclassification index (NRI) and integrated discrimination improvement (IDI), while the model calibration was evaluated with the Brier score. A p-value of <0.05 was considered statistically significant. Confidence intervals for evaluation metrics were estimated using the bootstrap method with 1,000 resamples.

## Results

### Patient and nodule characteristics

A total of 514 participants were recruited. Among all participants, 1 participant had no pathological report, 3 participants had no peripheral blood sample, and 9 participants had no compliant CT image. Finally, 501 participants with 571 GGNs were included in this study. Four hundred and seven participants with 454 GGNs were assigned to the training-validation group, whereas 94 participants with 117 GGNs were assigned to the test group (**Figure 1**).

There were 307 female (61.3%) and 194 male (38.7%) participants, and the median age was 57.0 years. There were 216 pure (37.8%) and 355 mixed (62.2%) GGNs, and the median size was 11.8 mm. Pathologically, there were 59 benign (10.3%) and 512 malignant (89.7%) GGNs. The pathologies of the benign GGNs included chronic inflammation (n=35), fibrous hyperplasia (n=12), organizing pneumonia (n=3), sclerosing pneumocytoma (n=1), pleomorphic adenoma (n=1), and atypical adenomatous hyperplasia (n=7). The pathologies of the malignant GGNs included adenocarcinoma *in situ* (n=37), minimally invasive adenocarcinoma (n=132), invasive adenocarcinoma (n=339), squamous cell carcinoma (n=3), and lymphoma (n=1).

Statistical analysis revealed significant differences between participants with and without malignancies in sex, smoking history, TPI-1, and miR-206 (**Table 1**). Additionally, benign and malignant GGNs exhibited significant differences in size, location, and lobulation (**Table 2**).

**Table 1:** Characteristics of participants included in this study.

	Total	Without Malignancy	With Malignancy	*p*
	(n = 501)	(n = 37)	(n = 464)	
Sex:				**0.030**
Female	307 (61.3%)	16 (43.2%)	291 (62.7%)	
Male	194 (38.7%)	21 (56.8%)	173 (37.3%)	
Age	57.0 [49.0;64.0]	52.0 [46.0;61.0]	57.0 [49.0;64.0]	0.142
Smoking history	112 (22.4%)	16 (43.2%)	96 (20.7%)	**0.003**
Personal tumor history	23 (4.59%)	1 (2.70%)	22 (4.74%)	1.000
Family lung tumor history	51 (10.2%)	4 (10.8%)	47 (10.1%)	0.782
CEA (μg/L)	1.73 [1.12;2.61]	1.51 [1.30;1.94]	1.74 [1.09;2.69]	0.363
TPI-1 (ng/L)	270 [215;335]	245 [190;300]	270 [220;340]	**0.023**
miR-206	2.31 [0.90;7.19]	1.20 [0.30;4.06]	2.40 [0.95;7.70]	**0.015**

The bold values indicate p<0.05.

**Table 2:** Characteristics of GGNs included in this study.

	Total	Benign	Malignant	*p*
	(n = 571)	(n = 59)	(n = 512)	
Size (mm)	11.8 [9.15;17.0]	10.0 [7.45;17.4]	12.0 [9.30;17.0]	**0.020**
Location:				0.033
Left lower lobe	76 (13.3%)	5 (8.47%)	71 (13.9%)	
Left upper lobe	151 (26.4%)	14 (23.7%)	137 (26.8%)	
Right lower lobe	100 (17.5%)	16 (27.1%)	84 (16.4%)	
Right middle lobe	54 (9.46%)	10 (16.9%)	44 (8.59%)	
Right upper lobe	190 (33.3%)	14 (23.7%)	176 (34.4%)	
Type:				0.367
pGGN	216 (37.8%)	26 (44.1%)	190 (37.1%)	
mGGN	355 (62.2%)	33 (55.9%)	322 (62.9%)	
Lobulation	106 (18.6%)	3 (5.08%)	103 (20.1%)	**0.008**
Spiculation	53 (9.28%)	2 (3.39%)	51 (9.96%)	0.158
Vacuole	65 (11.4%)	3 (5.08%)	62 (12.1%)	0.164
Pleural retraction	82 (14.4%)	4 (6.78%)	78 (15.2%)	0.119
Vascular convergence	38 (6.65%)	7 (11.9%)	31 (6.05%)	0.098
Air bronchogram	18 (3.15%)	0 (0.00%)	18 (3.52%)	0.241
Well-defined boundary	510 (89.3%)	53 (89.8%)	457 (89.3%)	1.000

The bold values indicate p<0.05.

### Model construction

**Figure 3:**
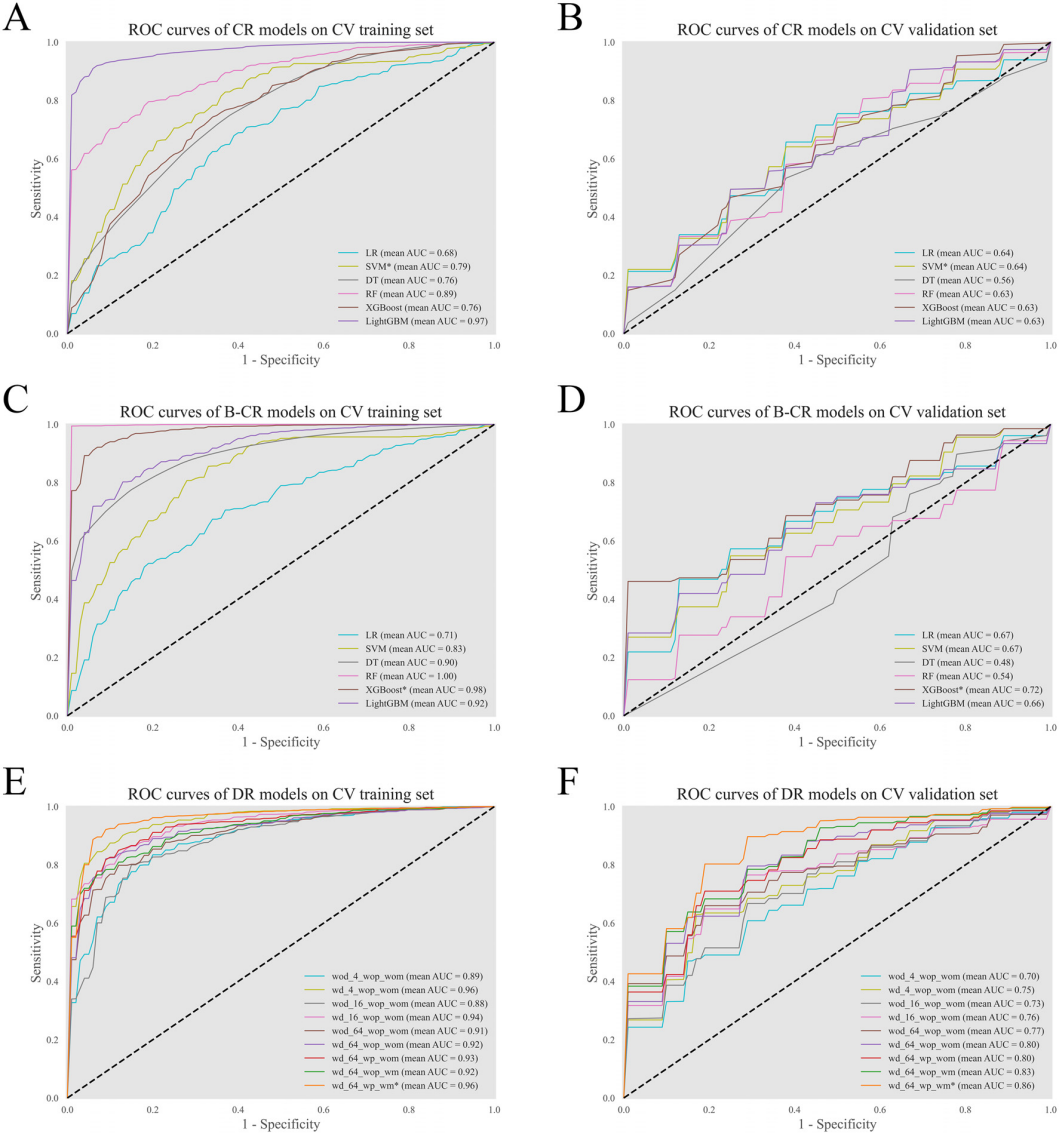
Performance of the models in fivefold cross-validation. The ROC curves of the CR models **(A, B)**, the B-CR models **(C, D)**, and the DR models **(E, F)** on the training set and the validation set. For the DR models, the tag wd/wod specifies whether the model used the distance map as an additional input; the tag wp/wop denotes whether the model was initialized with pretrained weights; the tag wm/wom refers to whether the model employed the manifold mixup technique during training. ^*^The optimal model.

The clinical-radiological (CR) models employed sex, smoking history, size, and lobulation as input features. Based on the cross-validation results (**Figure 3**), the optimal CR model was developed using the SMV algorithm. The biomarker-combined CR (B-CR) models incorporated additional biomarkers, TPI-1 and miR-206. And the final B-CR model was constructed with the XGBoost algorithm.

For the deep radiomic (DR) models, cross-validation results indicated a positive correlation between model complexity and performance. And incorporating the distance map improved the performance of models across all complexity levels. Further experiments demonstrated that the manifold mixup technique also contributed to performance enhancement. Notably, the pretraining strategy enhanced model performance only when applied in conjunction with the mixup technique. Based on these findings, the final DR model was constructed using the most complex model architecture. And the distance map, the manifold mixup technique, and the pretraining strategy were all applied during the model construction.

The clinic-combined DR (C-DR) model, the biomarker-combined DR (B-DR) model, and the clinic-biomarker-combined DR (CB-DR) model were established using the same hyperparameters and settings of the final DR model.

### Model evaluation

**Figure 4:**
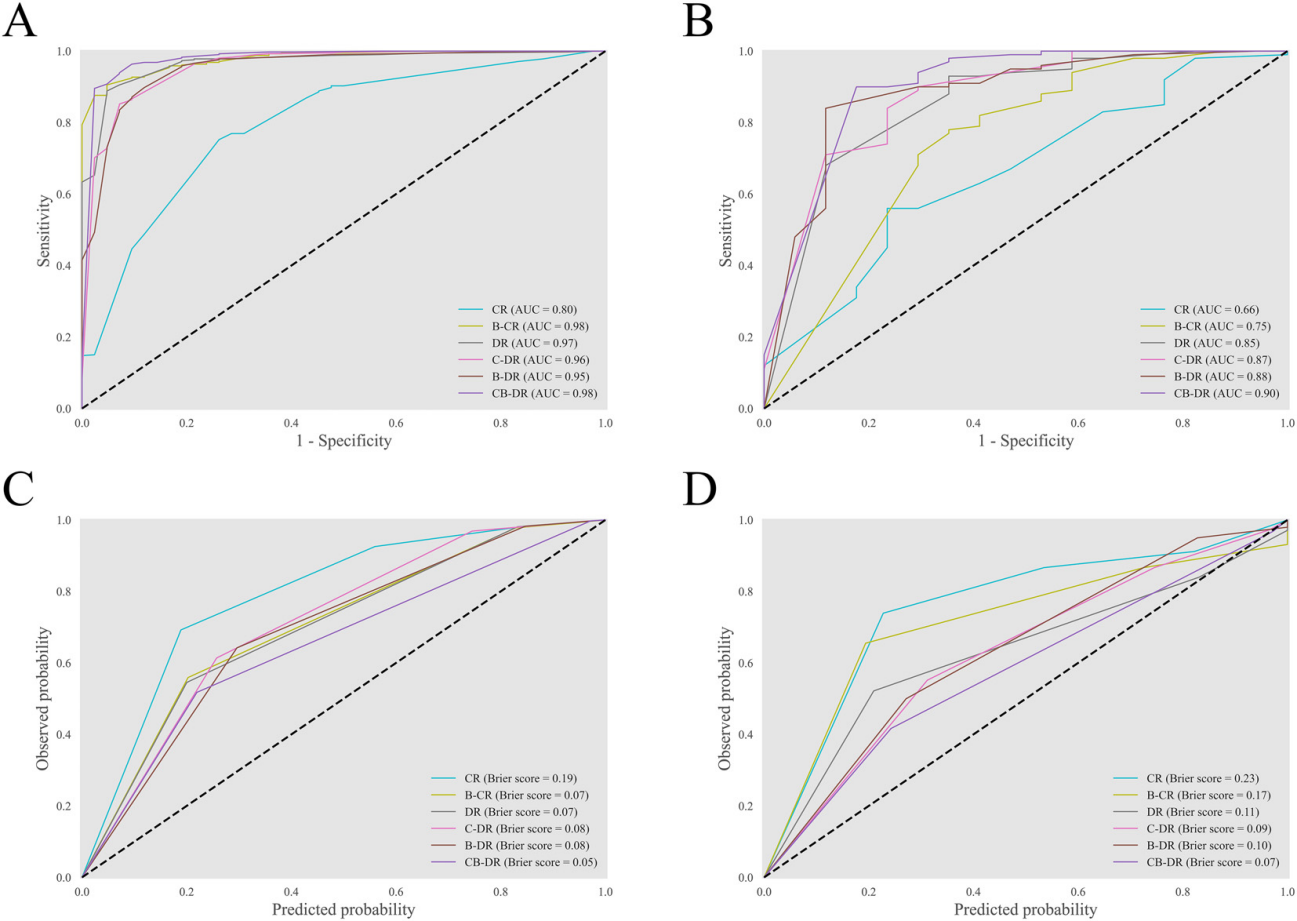
Discrimination and calibration of the models. **(A, B)** The ROC curves of the models on the training-validation set and the test set. **(C, D)** The reliability diagrams of the models on the training-validation set and the test set.

The discrimination of the models was evaluated (**Figures 4A, B**). The CR model had an AUC of 0.66 (95% CI: 0.51–0.79). After incorporating biomarkers, the AUC of the B-CR model increased to 0.75 (95% CI: 0.60–0.88). And the IDI and NRI of the B-CR model compared to the CR model were 0.235 and 0.190, respectively. Similarly, the AUC of the C-DR model, the B-DR model, and the CB-DR models was higher than that of the DR model. The corresponding IDI and NRI were 0.004, 0.052, and 0.139, and 0.078, 0.079, and 0.196, respectively. These results suggested that the multimodal models provided better discrimination between benign and malignant GGNs. Among all the models, the CB-DR model achieved the highest AUC of 0.90 (95% CI: 0.81–0.97). At the optimal cutoff, the accuracy, sensitivity, and specificity of the CB-DR model were 0.89 (95% CI: 0.83–0.94), 0.90 (95% CI: 0.84–0.96), and 0.82 (95% CI: 0.62–1.00), respectively (**Table 3**). This indicated that the CB-DR model could provide an accurate basis for clinical decision-making.

**Table 3:** Classification metrics of the models.

Model	AUC	Accuracy	Sensitivity	Specificity
CR:				
Training-validation set	0.80 [0.73, 0.86]	0.75 [0.71, 0.79]	0.75 [0.71, 0.79]	0.74 [0.60, 0.87]
Test set	0.66 [0.52, 0.80]	0.62 [0.54, 0.71]	0.63 [0.54, 0.72]	0.59 [0.33, 0.82]
B-CR:				
Training-validation set	0.98 [0.96, 0.99]	0.91 [0.88, 0.94]	0.91 [0.88, 0.93]	0.95 [0.88, 1.00]
Test set	0.75 [0.59, 0.87]	0.79 [0.71, 0.86]	0.82 [0.74, 0.89]	0.59 [0.35, 0.82]
DR:				
Training-validation set	0.97 [0.94, 0.99]	0.89 [0.87, 0.92]	0.89 [0.86, 0.92]	0.95 [0.88, 1.00]
Test set	0.85 [0.74, 0.94]	0.85 [0.79, 0.91]	0.88 [0.82, 0.94]	0.65 [0.41, 0.87]
C-DR:				
Training-validation set	0.96 [0.92, 0.98]	0.86 [0.83, 0.89]	0.85 [0.82, 0.89]	0.93 [0.85, 1.00]
Test set	0.87 [0.77, 0.96]	0.83 [0.75, 0.89]	0.84 [0.76, 0.91]	0.76 [0.54, 0.94]
B-DR:				
Training-validation set	0.95 [0.91, 0.98]	0.90 [0.87, 0.92]	0.90 [0.87, 0.92]	0.88 [0.77, 0.97]
Test set	0.88 [0.77, 0.97]	0.87 [0.80, 0.92]	0.90 [0.84, 0.96]	0.71 [0.46, 0.91]
CB-DR:				
Training-validation set	0.98 [0.95, 1.00]	0.90 [0.87, 0.93]	0.90 [0.87, 0.92]	0.98 [0.92, 1.00]
Test set	0.90 [0.81, 0.98]	0.89 [0.83, 0.94]	0.90 [0.84, 0.96]	0.82 [0.62, 1.00]

The reliability diagram was plotted to visualize the consistency between the predicted malignancy probabilities and the actual observations (**Figures 4C, D**). The diagrams revealed that all the models underestimated the malignancy risk of GGNs to varying degrees. Among all the models, the multimodal models showed relatively better calibration. The CR model exhibited the most significant underestimation, with a Brier score of 0.23 (95% CI: 0.20–0.27). In contrast, the CB-DR model demonstrated the best calibration and achieved a Brier score of 0.07. Additionally, the Spiegelhalter’s z-test indicated no significant difference between the predictions by the CB-DR model and the observed outcomes (*p*=0.23). These results indicated that the CB-DR model was a properly calibrated model.

**Figure 5:**
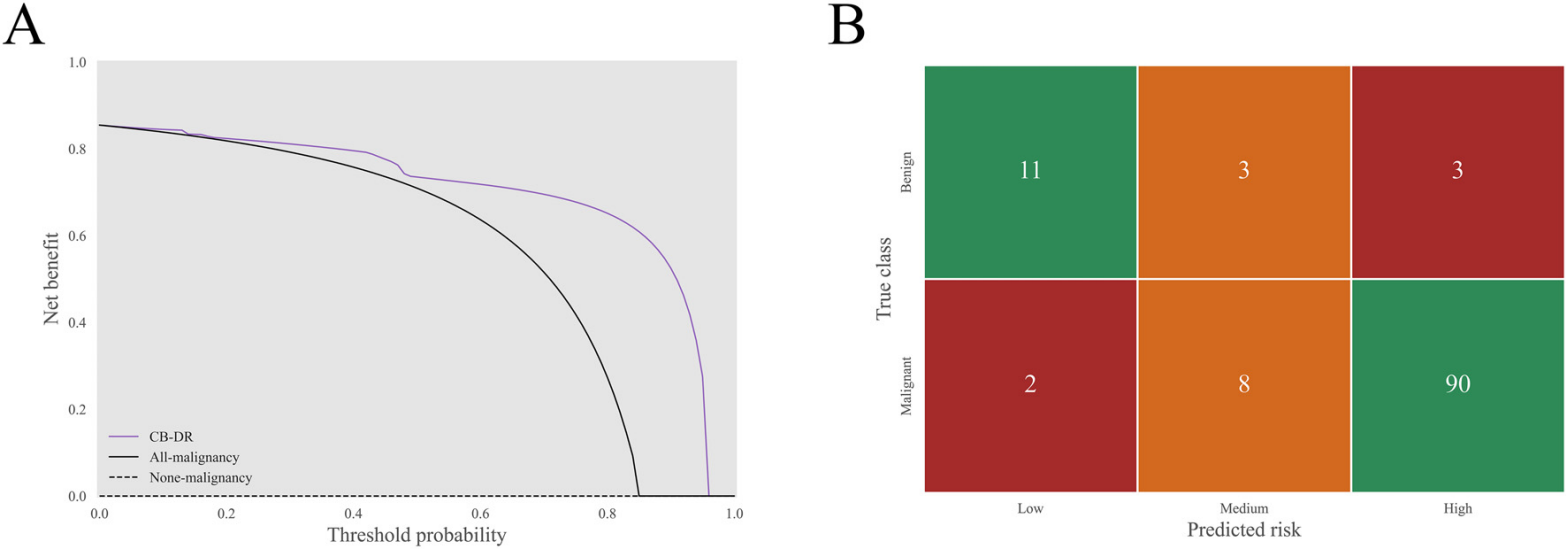
Clinical utility of the CB-DR model. **(A)** The decision curves of the CB-DR model on the test set. In a threshold probability range of 0.00 to 0.96, the CB-DR model exhibited a greater net benefit than the all-malignancy and none-malignancy reference. **(B)** The corresponding stratification matrix of the CB-DR model. The CB-DR model stratified 2.0% (2/100), 8.0% (8/100), and 90.0% (90/100) of malignant GGNs and 64.7% (11/17), 17.7% (3/17), and 17.7% (3/17) of benign GGNs into the low-, medium-, and high-risk groups.

The CB-DR model was further evaluated for its clinical utility. The decision curves were plotted to analyze the net benefit under different threshold probabilities (**Figure 5A**). The curves showed that the CB-DR model had a greater net benefit than the all-malignancy and none-malignancy reference when the threshold probability was between 0.00 and 0.96. This suggested that the CB-DR model had a wide range of clinical utility.

To improve clinical applicability, the sensitivity-preferred (for high sensitivity) and specificity-preferred (for high specificity) cutoff values, set at 0.16 and 0.95 respectively, were applied to stratify GGNs into low-, medium-, and high-risk. On the basis of this risk stratification, the distributions of low-, medium-, and high-risk were 2.0% (2/100), 8.0% (8/100), and 90.0% (90/100) in malignant GGNs, and 64.7% (11/17), 17.7% (3/17), and 17.7% (3/17) in benign GGNs (**Figure 5B**). In a hypothetical clinical management framework, the following strategies could be implemented based on risk levels: low-risk GGNs would undergo extended-interval follow-up, medium-risk GGNs would receive routine surveillance, and high-risk GGNs would be subject to invasive intervention. In this simulated clinical decision scenario, the CB-DR model demonstrated the potential to reduce overtreatment for 82.4% (14/17) of benign GGNs and enable timely interventions for 90.0% (90/100) of malignant GGNs.

**Figure 6:**
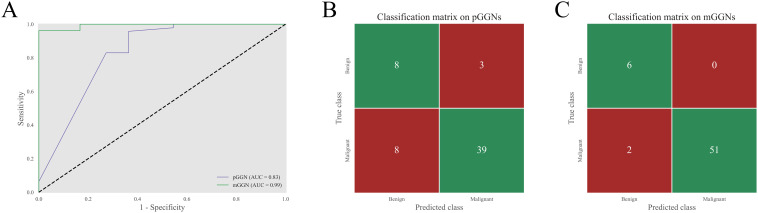
Performance of the CB-DR model for GGNs of different types. **(A)** The ROC curves of the CB-DR model on pGGNs and mGGNs in the test set. **(B, C)** The corresponding classification matrices of the CB-DR model on pGGNs and mGGNs. The overall accuracy of the CB-DR model on pGGNs and mGGNs were 81.0% (47/58) and 96.6% (57/59), respectively. For pGGNs, the CB-DR model correctly classified 72.7% (8/11) of benign pGGNs and 83.0% (39/47) of malignant pGGNs. And for mGGNs, the CB-DR model achieved correct classification for 100.0% (6/6) benign mGGNs and 96.2% (51/53) of malignant mGGNs.

Furthermore, the predictive performance of the CB-DR model for GGNs of different types was assessed (**Figure 6**). The results indicated that the CB-DR model performed better in predicting the malignancy of mGGNs compared to pGGNs (AUC: 0.99 vs. 0.83). The CB-DR model correctly classified 81.0% (47/58) of pGGNs and 96.6% (57/59) of mGGNs. Specifically, the model achieved correct classification for 72.7% (8/11) of benign pGGNs and 83.0% (39/47) of malignant pGGNs, whereas 100.0% (6/6) benign mGGNs and 96.2% (51/53) of malignant mGGNs were correctly classified by the model.

### Prediction demonstration

Two GGNs were selected to demonstrate the predictions by the CB-DR model. The first GGN was a benign pGGN with a diameter of 22.0 mm. It was from a 52-year-old female participant with no smoking history. Her serum TPI-1 concentration and relative expression of miR-206 were 250.0 ng/L and 1.2, respectively. The second GGN was a malignant pGGN with a diameter of 10.8 mm. It was from a 30-year-old female participant with no smoking history. The values of TPI-1 and miR-206 of the participant were 300.0 ng/L and 159.0, respectively. The CB-DR model correctly predicted that the first GGN was a medium-risk benign GGN with malignancy risk of 0.54, and the second GGN was a high-risk malignant GGN with malignancy risk of 0.97.

**Figure 7:**
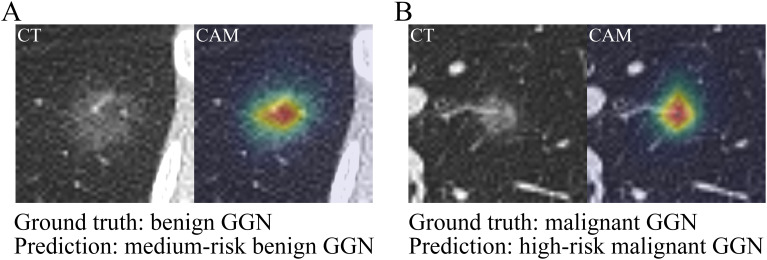
Prediction demonstration of the CB-DR model. The CT images and CAM diagrams of two selected GGNs. **(A)** The first GGN was a benign pGGN with a diameter of 22.0 mm. The CB-DR model predicted that the GGN was a medium-risk benign GGN with malignancy risk of 0.54. **(B)** The second GGN was a malignant pGGN with a diameter of 10.8 mm. The CB-DR model predicted that the GGN was a high-risk malignant GGN with malignancy risk of 0.97.

The CAM diagrams were generated to explain the visual basis of the CB-DR model for the predictions (**Figure 7**). The diagrams showed that the model was activated mainly by the voxels located in the GGNs. The voxels around the GGNs also activated the model to some extent. However, the voxels in the other regions had little effect on the model. This indicated that the predictions were based on voxels in reasonable regions.

## Discussion

Accurate classification of malignant and benign GGNs is essential for the effective clinical management of GGNs. At present, the evaluation of GGN malignancy primarily depends on dynamic radiological follow-ups. However, GGNs tend to progress slowly and possibly require surveillance over a span of 5–10 years (5). This prolonged period of diagnostic uncertainty, coupled with frequent CT follow-ups, can place significant psychological, social, and financial burdens on patients, increasing the risk of overdiagnosis and overtreatment (13, 14). Therefore, the development of accurate noninvasive methods for accurately classifying GGNs is crucial.

Previous studies on predicting the malignancy of GGNs have primarily relied on unimodal or bimodal features. For example, Zhang et al. developed a predictive model based on handcrafted radiomic features, achieving an AUC of 0.73 (26). Liang et al. integrated clinical and radiomic features to predict the malignancy of GGNs, with an AUC of 0.70 (27). And Huang et al. utilized a deep radiomics approach to construct a predictive model, achieving an AUC of 0.89 (28). In this study, we developed and validated a predictive model for the malignancy of GGNs, the CB-DR model. The model exhibited satisfactory performance and achieved an AUC of 0.90 (95% CI: 0.81-0.97). The performance of the model is at an advanced level compared to similar studies. It correctly classified 82.4% of the benign GGNs and 90.0% of the malignant GGNs. And clinical decision-making supported by the CB-DR model would have reduced risks of overdiagnosis and overtreatment.

Radiological features provide significant convenience and accessibility and play an important role in the clinical management of GGNs. Previous studies indicated that large size, mGGN, lobulation, spiculation, vacuole, and well-defined margin are associated with malignant GGNs (29). In this study, we observed similar findings; however, only size and lobulation exhibited significant differences between benign and malignant GGNs. Notably, compared to solid nodules, GGNs have a lower positive rate for radiological signs (26). In our study, the positive rates for radiological signs ranged from 5% to 20%. This partially limits the predictive performance of radiological features and their applicability in GGN management. Additionally, recent studies have demonstrated that parameters derived from PET/CT hold certain diagnostic value for malignant GGNs (30, 31). However, considering economic constraints and clinical feasibility, our study employed CT imaging, which is more widely accessible and routinely used in clinical practice, to predict the malignancy of GGNs.

With the deepening understanding of the molecular mechanisms underlying lung cancer and the development of advanced biomolecular detection technologies, an increasing number of studies have explored the diagnostic potential of serum proteins and miRNAs in lung cancer (32, 33). In our previous research, we identified two biomarkers, TPI-1 and miR-206, with potential value in differentiating malignant and benign GGNs. TPI-1 is an enzyme involved in glycolysis that catalyzes the conversion of dihydroxyacetone phosphate (DHAP) to glyceraldehyde 3-phosphate (GAP). Prior studies have shown that TPI-1 is overexpressed in lung cancer and plays a key role in tumorigenesis (34). And miR-206 was initially identified as a muscle-specific microRNA; however, subsequent research has suggested its association with lung cancer, although its role in lung cancer remains inconclusive (35). In this study, we observed significant differences in TPI-1 and miR-206 between patients with or without GGNs. Furthermore, combining these biomarkers with other features improved the predictive performance for the malignancy of GGNs. To the best of our knowledge, no studies have previously reported the use of TPI-1 in the differentiation of malignant and benign pulmonary nodules. For miR-206, Liu’s study suggested that it may serve as a biomarker for the early diagnosis of lung cancer (36). However, Liu reported a decreased relative expression of miR-206 in the serum of lung cancer patients. This contradicts our findings. This discrepancy is likely due to substantial heterogeneity between the study populations. Liu’s research focused on elderly male smokers, a group not typically associated with GGNs, whereas our study involved primarily younger female nonsmokers.

Deep radiomics offers significant advantages in the adaptive extraction of imaging features. In our study, the DR model outperformed the CR model. The integration of clinical features and biomarkers further improved the performance. This indicated the robust capability of deep radiomics to learn complex patterns. However, deep radiomics generally requires large datasets for optimal performance. To mitigate the risk of overfitting due to the limited sample size, we set the candidate gradient for model complexity starting at a low level. Surprisingly, models with higher complexity performed better. In the training of deep models, regularization is typically employed by default to prevent overfitting. The AdamW optimizer used in this study for training employed L-2 regularization, which likely helped minimize the impact of overfitting in our deep radiomic analysis. Thus, rather than overfitting, the ability to extract superior features, potentially through more complex models, may have a greater influence on performance. Previous studies have shown that using complete image patches improved model performance by incorporating microenvironmental information surrounding the nodules (17). However, GGNs are relatively difficult to be identified accurately by deep models (37). We suspected that using only complete image patches might cause models to fit too many features from irrelevant regions. Therefore, the distance map was considered in this study as an additional input to guide models to focus on relevant regions. Our results show that this is an effective method to improve performance. Moreover, we utilized the manifold mixup technique and the pretraining strategy to reduce the reliance on large datasets. Our findings showed that these training tricks effectively enhanced performance with the limited sample size. For deep radiomic models, both model architectures and appropriate training techniques are crucial to achieving optimal performance.

Different modalities, such as clinical features, CT images, and biomarkers, provide unique types of information and generally capture only specific aspects of lesions. Therefore, unimodal data may have limitations in reflecting the full spectrum of lesion characteristics. Multimodal data can compensate for these limitations and enable models to capture a more comprehensive understanding of pathology. In this study, we integrated clinical, biomarker, and deep radiomic features to construct a multimodal model. The model exhibited superior classification performance. Previous studies similarly demonstrated that multimodal features increased model performance (20). In their study, incorporating DNA methylation biomarkers into clinical and radiological features increased the AUC of their model from 0.85 to 0.90. Thus, multimodal approaches may represent a promising direction.

This study has several limitations. First, the sample size was limited. Second, to ensure accurate labeling of GGNs, we included only GGNs with confirmed pathological results, which resulted in a dataset predominantly composed of malignant GGNs. Although we employed data augmentation, pretraining, and other techniques to mitigate these limitations, they might still represent bottlenecks to performance. Finally, GGN segmentation in this study was performed manually. Manual segmentation is time-consuming, limits clinical applicability, and introduces a degree of subjectivity. We are currently conducting another clinical study on GGNs, and in future research, we plan to expand the sample size, increase the proportion of benign nodules, and develop an automated GGN segmentation model to further refine our current CB-DR model.

## Conclusion

This study developed and validated a clinic-biomarker-combined deep radiomic model to predict the malignancy of GGNs. The model demonstrated satisfactory performance and shows potential as a valuable tool for assisting clinical decision-making in the management of GGNs.

## Data Availability

The private dataset used in this study is not readily available due to restrictions imposed by ethical guidelines and data privacy regulations. Requests to access the dataset should be directed to Liang-an Chen at lianganchen301@263.net.
