# Supplementary material for: Integrating multimodal features to predict the malignancy of pulmonary ground−glass nodules: a multicenter prospective model development and validation study
Source: Front Oncol. 2025 Mar 21;15:1547816. doi: 10.3389/fonc.2025.1547816 (PMC11968343; doi:10.3389/fonc.2025.1547816)
Supplement: Supplementary file 1 [file DataSheet1.docx]

Supplementary Material

#
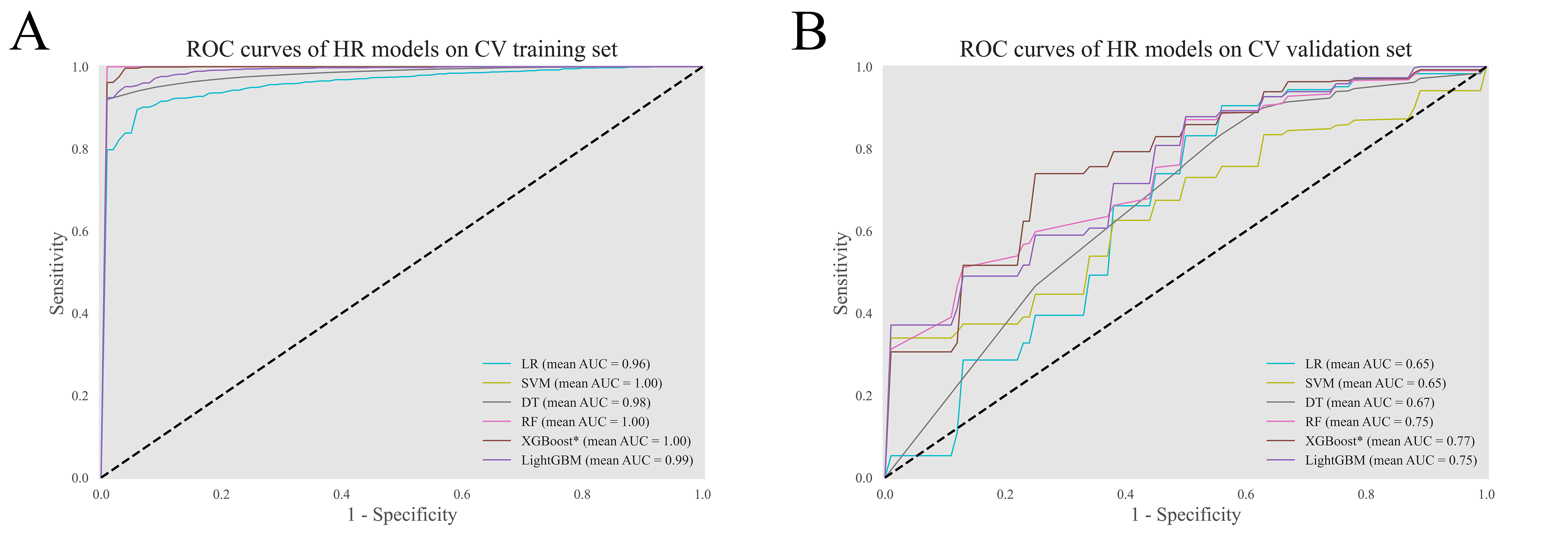
Supplementary Figures


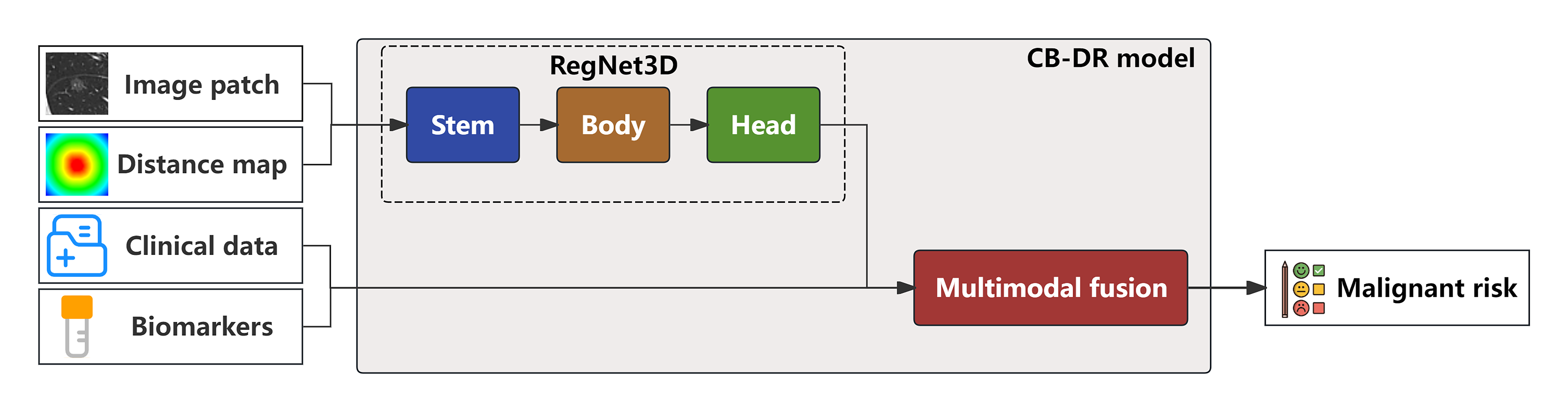
**Supplementary Figure 1.** Performance of the handcrafted radiomic (HR) models in fivefold cross-validation. The ROC curves of the HR models on the training set (A) and the validation set (B). We evaluated the applicability of handcrafted radiomics in predicting the malignancy risk of GGNs. A total of 1,223 HR features were extracted from each nodule using PyRadiomics (version 3.1.0; radiomics.io) (1). PyCaret was used for model development. The results indicated that the XGBoost-based HR model achieved the highest predictive performance, with an average AUC of 0.77 in fivefold cross-validation on the validation set; however, it was outperformed by the optimal deep radiomic model, which achieved an average AUC of 0.86. Consequently, deep radiomics was selected as the preferred method for radiomics modeling. ^*^The optimal model.

**Supplementary Figure 2.** Schematic diagram of the CB-DR model. The CB-DR model consists of a RegNet module and a multimodal fusion module. Deep radiomic features are extracted from the CT image and the corresponding distance map using the RegNet module. Subsequently, the multimodal fusion module integrates features from different modalities through a concatenation operation. Finally, the fused features are processed by a linear layer to calculate the malignancy risk of GGNs.

# Supplementary Tables

Supplementary Table 1. Regression metrics of the DR models. Mean absolute errors (MAEs) of pretrained models for different scores on the training set and the validation set. The tag wd specifies that the model used the distance map as an additional input. The tag wm/wom refers to whether the model uses the manifold mixup technique during training.

| **Model** | **Margin** | **Lobulation** | **Spiculation** | **Malignancy** |
| --- | --- | --- | --- | --- |
| wd_64_wom: |  |  |  |  |
| Training set | 0.33 [0.31, 0.35] | 0.37 [0.35, 0.40] | 0.32 [0.30, 0.34] | 0.32 [0.30, 0.33] |
| Test set | 0.35 [0.31, 0.39] | 0.42 [0.37, 0.48] | 0.41 [0.35, 0.47] | 0.36 [0.32, 0.40] |
| wd_64_wm: |  |  |  |  |
| Training set | 0.34 [0.32, 0.36] | 0.38 [0.36, 0.40] | 0.31 [0.29, 0.33] | 0.33 [0.31, 0.34] |
| Test set | 0.38 [0.34, 0.42] | 0.43 [0.38, 0.48] | 0.40 [0.34, 0.46] | 0.37 [0.33, 0.41] |

**References**

1. Van Griethuysen JJM, Fedorov A, Parmar C, Hosny A, Aucoin N, Narayan V, Beets-Tan RGH, Fillion-Robin J-C, Pieper S, Aerts HJWL. Computational Radiomics System to Decode the Radiographic Phenotype. *Cancer Research* (2017) 77:e104–e107. doi: 10.1158/0008-5472.CAN-17-0339
